# Complete Genome Sequence Assembly and Annotation for Myxococcus xanthus Strains DK1050 and DK101

**DOI:** 10.1128/mra.01020-22

**Published:** 2023-02-07

**Authors:** Alfonso López-Rojo, Diego Bernal-Bernal, S. Padmanabhan, María Luisa Galbis-Martínez, Montserrat Elías-Arnanz

**Affiliations:** a Departamento de Genética y Microbiología, Área de Genética (Unidad Asociada al IQFR-CSIC), Universidad de Murcia, Murcia, Spain; b Departamento de Química-Física Biológica, Instituto de Química Física Rocasolano, Madrid, Spain; Montana State University

## Abstract

Myxococcus xanthus is a social Gram-negative soil bacterium and the best studied member of the order *Myxococcales* in the class Deltaproteobacteria, which was recently reclassified as the phylum *Myxococcota*. Here, we report complete genomes, obtained using Illumina and PacBio sequencing, of M. xanthus strains DK1050 and DK101 (GenBank accession numbers CP104804 and CP104803, respectively).

## ANNOUNCEMENT

The Gram-negative soil bacterium Myxococcus xanthus is the best studied member of the myxobacteria, a phylogenetically distinct bacterial group constituting the order *Myxococcales* in the class Deltaproteobacteria (1, 2) but recently proposed for reclassification as a separate phylum, *Myxococcota* (3). Myxobacteria are evolutionarily advanced, with complex lifestyles and various eukaryote-type traits such as multicellular development, social behavior, motility, kin recognition, predation, and biosynthesis of specialized lipids and steroids (4–8). Genome sequencing and assembly were reported first for the widely used M. xanthus laboratory strain DK1622 (9) and subsequently for some other strains. Here, we report the complete genome sequence assembly and annotation for strains DK1050 and DK101. DK1050 facilitated the identification of DKxanthenes, the yellow M. xanthus pigments that are essential for mature spore formation in fruiting body development (10), and several new protein families and functions from studies of light-induced carotenogenesis (11), such as B_12_-based photoreceptors (12, 13) and a long-sought desaturase for human plasmalogen biosynthesis (7, 14). DK1050 (15) and social-motility-deficient DK101 (the precursor of the fully motile DK1622) were first described as single-colony isolates from a mixture of related strains denominated FB (16).

M. xanthus DK1050 and DK101 cells from our laboratory cryostock were grown at 33°C in 10 mL Casitone-Tris (CTT) buffer (1% Casitone, 8 mM MgSO_4_, 10 mM Tris-HCl, 1 mM potassium phosphate [pH 7.6] [17]) and harvested from 3 mL by centrifugation. Genomic DNA was extracted using the Wizard genomic DNA purification kit (Promega). The final precipitated DNA was dissolved overnight in sterile water at 4°C, and its quality and quantity were assessed using a Nanodrop spectrophotometer (Thermo Fisher Scientific) and a Qubit3 fluorometer (Invitrogen). Library preparation, quality control, sequencing, and assembly were performed by Novogene (UK) using their standard operating procedures. Briefly, libraries for short reads were constructed using genomic DNA that had been randomly sheared to ~350-bp fragments and a NEBNext DNA library preparation kit (strictly adhering to kit protocols for end repair, dA-tailing, adapter ligation, and PCR enrichment), purified, quality checked, and sequenced using an Illumina NovaSeq system (150-bp paired-end reads). Raw Illumina reads were filtered by discarding reads with adapter contamination, >10% uncertain nucleotides, or >50% low-quality nucleotides (Q scores of ≤5) and mapped with Novogene in-house software. Long reads from the Pacific Biosciences (PacBio) Sequel II system using single-molecule real-time SMRTbell libraries (PacBio, CA) were used for *de novo* assembly and resolution of repetitive and complex genomic regions. Short reads facilitated assessment of long-read accuracy and identification of variants relative to the DK1622 genome (GenBank assembly accession number GCA_000012685.1). Genomes were assembled using Novogene HGAP software and the Arrow algorithm in the final assembly polishing step and were annotated with NCBI Prokaryotic Genome Annotation Pipeline (PGAP) v6.2 (18). Default parameters were used for all software unless otherwise noted. Table 1 summarizes the results of the sequencing, assembly, and annotation of the circular DK1050 and DK101 genomes, rotated to the available DK1622 start position, and comparison with DK1622.

**TABLE 1:** M. xanthus DK1050 and DK101 genome assemblies and comparison with DK1622

Characteristic^*a*^	Data for strain:		
	DK1050 (GenBank accession no. CP104804)	DK101 (GenBank accession no. CP104803)	DK1622^*b*^ (GenBank accession no. CP000113.1)
Genome size (bp)	9,253,193	9,346,413	9,139,763
GC content (%)	68.80	68.77	68.89
Total no. of genes	7,508	7,602	7,364
Total no. of CDSs	7,426	7,520	7,282
No. of coding genes	7,342	7,433	7,199
No. of CDSs with protein	7,342	7,433	7,199
No. of RNA genes	82	82	82
No. of rRNAs (5S, 16S, 23S)	4, 4, 4	4, 4, 4	4, 4, 4
No. of tRNAs	66	66	66
No. of ncRNAs	4	4	4
No. of CDSs without protein	84	87	83
Total no. of pseudogenes	84	87	83
No. with ambiguous residues	0	0	0
No. frameshifted	21	22	19
No. incomplete	73	74	72
No. with internal stop	11	12	10
No. with multiple problems	19	19	16
No. of CRISPR arrays	4	4	4
No. of regulatory RNAs (riboswitches)	7	7	7
No. of Mx alpha copies	2	3	1
Total no. of simple variants^*c*^	78	46	NA
No. nonsynonymous	38	19	
No. synonymous	26	15	
No. with frameshifts	2	2	
No. within intergenic regions	9	7	
No. of pseudogenes	3	3	
Illumina final coverage (×)	184	194	NA
No. of raw Illumina reads	7,225,058	7,774,748	NA
Illumina mapping rate (%)	99.08	98.93	NA
No. of PacBio subreads	263,475	197,953	NA
PacBio mean subread length (bp)	8,060	8,142	NA
PacBio mapping rate (%)	99.8	98.25	NA
PacBio total read length (bp)	2,123,826,239	1,611,755,401	NA

a CDS, coding DNA sequence; ncRNA, noncoding RNA.

b Previously reported (9) and deposited in GenBank. NA, not applicable.

c Relative to DK1622 and excluding the Mx alpha prophage region.

### Data availability.

Raw sequences were deposited in the NCBI SRA database under BioProject accession number PRJNA877085, and the complete DK1050 and DK101 genome sequences were deposited in NCBI GenBank under accession numbers CP104804 and CP104803, respectively.
